# Use of plant growth regulators to reduce 2-methyl-4-chlorophenoxy acetic acid-Na (MPCA-Na) damage in cotton (*Gossypium hirsutum*)

**DOI:** 10.1186/s12870-022-03917-x

**Published:** 2022-11-16

**Authors:** Quan-Cheng Zhang, Jing Wang, Jun-Gang Wang

**Affiliations:** College of Agriculture, Shihezi University, Shihezi, 832003 China

**Keywords:** *Gossypium hirsutum*, MCPA-Na, Physiological metabolism, Plant growth regulators, Protective enzymes, Yield

## Abstract

**Background:**

2-methyl-4-chlorophenoxy acetic acid-Na (MPCA-Na) is a phenoxy carboxylic acid selective hormone herbicide that is widely used in the crop fields. However, drift of MPCA-Na during application is highly damaging to cotton (*Gossypium hirsutum*) and other crop plants. This study was carried out from 2019 to 2020 to determine the effects of different concentrations of MPCA-Na on physiological and metabolic activities besides growth and yield of cotton plants at seedling, budding, flowering and boll stages. Moreover, we evaluated the different combinations of 24-epibrassinolide, gibberellin (GA_3_), phthalanilic acid and seaweed fertilizer to ameliorate herbicide damage.

**Results:**

2-methyl-4-chlorophenoxy acetic acid-Na (MPCA-Na) exposure caused a decrease in the chlorophyll content, and an increase in the soluble protein content, Malondialdehyde (MDA) content and protective enzyme activity. It also caused significant reductions in plant height, boll number and the single boll weight at the seedling and budding stages, but had little effects on plant height and the single boll weight at flowering and boll stage. Under the maximum recommended dose of MPCA-Na (130 g/L), the number of cotton bolls at seedling and budding stages decreased by 75.33 and 79.50%, respectively, and the single boll weight decreased by 46.42 and 36.31%, respectively. Nevertheless, the number of *G. hirsutum* bolls and single boll weight at flowering and boll stage decreased by 48.15 and 5.38%, respectively. Application of plant growth regulators decreased the MDA content, and increased chlorophyll, soluble protein content and protective enzyme activity, and alleviated MCPA-Na toxicity. Positive effects in case of growth regulators treated plants were also observed in terms of *G. hirsutum* yield. Phthalanilic acid + seaweed fertilizer, 24-epibrassinolide + seaweed fertilizer, and GA_3_ + seaweed fertilizer should be used at the seedling, budding, and flowering and boll stages, respectively.

**Conclusions:**

The results of current study suggest that certain plant growth regulators could be used to alleviate MPCA-Na damage and maintain *G. hirsutum* yield. When the cotton exposed to MCPA-Na at the seedling stage, it should be treated with phthalanilic acid + seaweed fertilizer, while plants exposed at the budding stage should be treated with 24-epibrassinolide + seaweed fertilizer, and those exposed at the flowering and boll stages should be treated with GA_3_ + seaweed fertilizer to mitigate stress.

**Supplementary Information:**

The online version contains supplementary material available at 10.1186/s12870-022-03917-x.

## Background

Cotton (*Gossypium hirsutum*) is an important economic crop in many places in the world [1]. In the process of cotton growth, often stress by pests and weeds. For a long time, the use of pesticides can reduce the harm of pests and weeds to cotton. However, in the use of pesticides, due to the nonstandard operation, pesticide damage often occurs in cotton, and it may occur at all growth stages of cotton [2]. Pesticide damage often causes cotton leaf deformity, chlorosis, and buds, flowers and young bolls were being prone to drop [3]. In particular, the herbicide damage has a great impact on the reduction of cotton yield. Therefore, plant growth regulators are widely used to alleviate and reduce the impact of herbicide damage on cotton, and are often used as remedial measures to reduce cotton yield loss [4].

2-methyl-4-chlorophenoxy acetic acid-Na (MCPA-Na) is a phenoxyacetic acid selective hormone herbicide [5]. It exposure disrupts the transportation tissue in dicotyledonous plants, interfering with plant growth and development, and thereby, achieving the purpose of weeding [6]. In China, MCPA-Na is widely used to control broad-leaf weeds such as pondweed, meadow pine, and sedge in wheat, rice, sugarcane and flax fields [7–10]. In addition, MCPA-Na is also used for inter-row directional weed control in the cotton fields [11]. However, due to the lack of a protective cover on the application nozzle, drift as well as direct exposure can also cause serious damage to the non target *G. hirsutum* plants [12]. Reducing the impact of cotton damage and the subsequent losses in yield is therefore essential.

For a long time, herbicide damage has been an important limiting factor of crop yield, especially in relation to inactivated herbicides [13–15]. Field observations show that cotton is extremely sensitive to MCPA-Na. Leaves of cotton plants exposed to this herbicide become pale and brittle as well as wrinkled and thickened with prominent veins, while the overall leaf shape becomes narrow, leaf margins of young leaves curl upwards, becoming cup-shaped, bracts turn red, and buds, flowers, and young bolls were being prone to drop [12]. Due to the inability of cotton to metabolize MCPA-Na, callus-like growths also develop on the lower stem and roots, resulting in the wilting of young buds and tissue necrosis [12]. Moreover, this damage often occurs during important growth periods, such as the seedling, bud, flowering or boll stages. In our previous study, exposure of cotton seedlings to MCPA-Na caused an increase in the soluble protein content and protective enzyme activity [13]. However, whether these modifications occur following MCPA-Na exposure during other growth stages and the subsequent effects on cotton yield remain unknown.

Plant growth regulators play an important role in regulating crop growth and development, improving yield and quality, and enhancing stress resistance [16, 17]. Notably, growth regulators improve the ability of plants to resist drought, salinity, and pests [18–22]. In addition, they are also used to alleviate pesticide damage. For example, brassinosteroids protects maize from amphetamine damage [21], while the combined application of sodium nitrophenolate, choline chloride and inositol reduce pyraclostrobin damage in soybean and maize seedlings [22]. Similarly, gibberellic acid was found to alleviate S-metolachlor damage in rice seedlings [23]. Recent research further suggests that plant growth regulators alleviate damage by improving the activity of protective enzymes [24]. However, their effectiveness in alleviating MCPA-Na toxicity in cotton is yet to be determined. Clarification of the detoxification mechanism of plant growth regulators in MCPA-Na-exposed *G. hirsutum* is therefore important.

During this study, we examined the effects of MCPA-Na on physiometabolic activities and yield traits in cotton following herbicide exposure at the seedling, budding, flowering and boll stages. Four plant growth regulators (24-epibrassinolide, GA_3_, phthalanilic acid and seaweed fertilizer) were used following MCPA-Na exposure, which could promote cotton plants growth. The protective effects of different combinations of plant growth regulators on MCPA-Na phytotoxicity were then examined at each growth stage. The results of this study provide an understanding of the underlying mechanism of MCPA-Na toxicity in *G. hirsutum*, supporting measures aimed at the use of plant growth regulators to alleviate damage, which has important practical significance for cotton producers. And the study provides a case for the solution of herbicide damage in other crops.

## Materials and methods

### Experimental field and plants

The field experiment was conducted at Shihezi University Educational Test Site, Xinjiang, China (86°E, 44°N). The soil was loam, containing 7.51 mg·kg^− 1^ organic carbon, 1.32 g·kg^− 1^ total nitrogen, 185 mg·kg^− 1^ available potassium and 7.12 mg·kg^− 1^ available phosphorus. The land has been growing cotton for more than 5 years. The experiment was conducted from 2019 to 2020.

The cotton variety used was Xinluzao 12 (provided by Institute of Cotton Research, Chinese Academy of Agricultural Sciences), and the seeds were treated with imidacloprid seed coating agent (Gaucho® Bayer Crop Science, 6 mL kg^-1^ seeds). Seeding was carried out under mulch film with width of 1.5 m. Row were spaced at (30 + 50 + 30) cm, the cotton plants were spaced above 10 cm, and with a planting density of 150,000 plants/hm^2^.

### Experimental field management

Before sowing, (NH_4_)_3_PO_4_ @ 175 kg hm^-^^2^ and urea @ 260 kg hm^-^^2^ were applied as basal fertilizer. The seeds were sown on 15 April, 2019 and 25 April, 2020. Irrigation was carried out four times throughout the growing period on 2019 (28 June, 18 July, 8 August, and 24 August) and 2020 (15 June, 7 July, 1 August, and 19 August), respectively. Drip irrigation was also carried out using 4125 and 4830 m^3^ hm^-^^2^ water on 2019 and 2020, respectively. During the cotton growth period (June–August), 525 kg hm^-^^2^ urea, and 450 kg hm^-^^2^ KH_2_PO_4_·NH_4_H_2_PO_4_ were also fertilization through drip irrigation. Chemical capping was carried out on 2019 (10 July and 25 July) and 2020 (3 July and 20 July) using 250–300 g hm^-^^2^ mepiquat chloride (Pix® HC, Basf), respectively. Pest and disease control were carried out according to the standard management practices, mainly to control cotton aphids, thrips and spider mites.

### MPCA-Na and plant growth regulators

The following plant growth regulators were used: 13% MPCA-Na aqueous solution (Songrun Pharmaceutical Factory, Jilin, China), 0.0075% 24-epibrassinolide aqueous solution (Xinchaoyang Crop Science Co., Ltd., Chengdu, China), 75% Gibberellin (GA_3_) crystal powder (Tongrui Biotechnology Co., Ltd., Shanghai, China), seaweed fertilizer (Xinhefeng Agrochemical Information Co., Ltd., Beijing, China), and 20% Phthalanilic acid soluble concentrate (Sunger Bioscience Co., Ltd., Shanxi, China).

### MCPA-Na application

According to the recommended dose of 13% MCPA-Na, concentrations of 0, 8.125, 16.25, 32.5, 65, and 130 g L^-1^ (500 kg water hm^-^^2^) were configured for use in this study. In the preparation of MCPA-Na with different concentrations, 8.125, 16.25, 32.5, 65, and 130 g MCPA-Na were weighed with electronic balance, respectively. Then, poured into a bucket containing 1 L of water, and stirred evenly with a glass rod. The prepared of MCPA-Na was poured into a 3WBD-20 L back-loaded electric sprayer. The preparation was repeated according to the above steps until the sprayer was filled with the MCPA-Na.

The experiment followed a completely randomized block design, with each plot measuring an area of 100 m^2^ (10 m × 10 m), which contained nine rows in the plot. A 3WBD-20 L back-loaded electric sprayer was used to evenly apply MCPA-Na at the seedling (growth of the fourth true leaf), budding, flowering and boll stages. Each treatment was repeated three times. Contents of chlorophyll, soluble protein and MDA, and activities of SOD, CAT and POD in the cotton leaves were then measured on days 1, 4, and 7 after treatment. Plant height, boll number and the single boll weight were also measured on 15 October 2019 and 10 October 2020 (harvest period) to determine the effect on yield.

### Application of plant growth regulators following MCPA-Na exposure

A complete randomized block design was also used to study the effects of the plant growth regulators, with each plot measuring 100 m^2^. MCPA-Na at the maximum concentration of 130 g L^-1^ was applied using the 3WBD-20 L back-loaded electric sprayer at the seedling, budding, flowering and boll stages. Two days after spraying MCPA-Na, different combinations of plant growth regulators were then applied (see Table 1 for each treatment combination). T0 (control), T1 (24-epibrassinolide), T2 (GA_3_ + seaweed fertilizer), T3 (24-epibrassinolide + seaweed fertilizer) and T4 (phthalanilic acid + seaweed fertilizer), T5 (GA_3_), T6 (24-epibrassinolide + GA_3_ + phthalanilic acid + seaweed fertilizer), T7 (phthalanilic acid), T8 (24-epibrassinolide + GA_3_ + phthalanilic acid).

**Table 1:** Combinations of plant growth regulators. '+' represents the addition of plant growth regulators, '-' represents the absence of plant growth regulators

	Gibberellin	Brassinosteroids	Phthalanilic acid	Seaweed fertilizer
T0	–	–	–	–
T1	–	+	–	–
T2	+	–	–	+
T3	–	+	–	+
T4	–	–	+	+
T5	+	–	–	–
T6	+	+	+	+
T7	–	–	+	–
T8	+	+	+	–

0.0075% 24-epibrassinolide, 75% gibberellin (GA_3_), 20% phthalanilic acid and seaweed fertilizer were applied at dosages of 0.25 mL L^-1^, 0.01 g L^-1^, 0.5 g L^-1^, and 3.5 mL L^-1^ (500 kg water hm^-^^2^), all of which represent the recommended concentrations of each component. Preparation of T1: 0.25 mL 24-epibrassinolide was poured into bucket filled with 1 L water. Preparation of T2: 0.01 g GA_3_ and 3.5 mL seaweed fertilizer were poured into bucket filled with 1 L water. Preparation of T3: 0.25 mL 24-epibrassinolide and 3.5 mL seaweed fertilizer were pour into bucket filled with 1 L water. Preparation of T4: 0.5 g phenylamino acid and 3.5 mL seaweed fertilizer were pour into bucket filled with 1 L water. Preparation of T5: 0.01 g GA_3_ was poured into bucket filled with 1 L water. Preparation of T6: 0.25 mL 24-epibrassinolide, 0.01 g GA_3_, 0.5 g phthalanilic acid and 3.5 mL seaweed fertilizer were poured into bucket filled with 1 L water. Preparation of T7: 0.5 g phenylamino acid was poured into bucket filled with 1 L water. Preparation of T8: 0.25 mL 24-epibrassinolide, 0.01 g GA_3_ and 0.5 g phthalanilic acid were poured into bucket filled with 1 L water. Each treatment was repeated three times. Contents of chlorophyll, soluble protein and MDA, and activities of SOD, CAT and POD in the cotton leaves were then measured as above on days 1, 4 and 7 after treatment. Plant height, boll number and the single boll weight were also measured on 15 October 2019 and 10 October 2020 (harvest period) to determine the effect on yield.

### Analysis of chlorophyll, soluble protein, MDA and protective enzyme activity in the cotton leaves

Chlorophyll content was measured using the acetone ethanol method [25], soluble protein was determined using the G-250 dye colorimetric method [26], and the MDA content was determined based on the thiobarbituric acid method [27].CAT, POD and SOD activity were determined using the guaiacol method [28], UV absorption method [29] and riboflavin-NBT method [30], respectively.

### Analysis of plant height, boll number and the single boll weight

On 15 October 2019 and 10 October 2020, 20 cotton plants were randomly selected from each treatment for analysis of plant height, boll number and the single boll weight. Plant height was determined as the length from the stem base to the growing point. The number of cotton bolls per plant, and the single boll weight was determined as the lint weight of a single cotton boll. Each treatment was repeated three times then mean values were obtained.

### Statistical analysis

Microsoft Excel 2010 was used for data processing and plotting. SPSS 20.0 data processing software was used for all statistical analyses, giving mean values and standard errors. The LSD test was used to test for significance between the differences in means.

## Results

### Effects of MCPA-Na exposure and application of plant growth regulators at the seedling stage

**Fig. 1:**
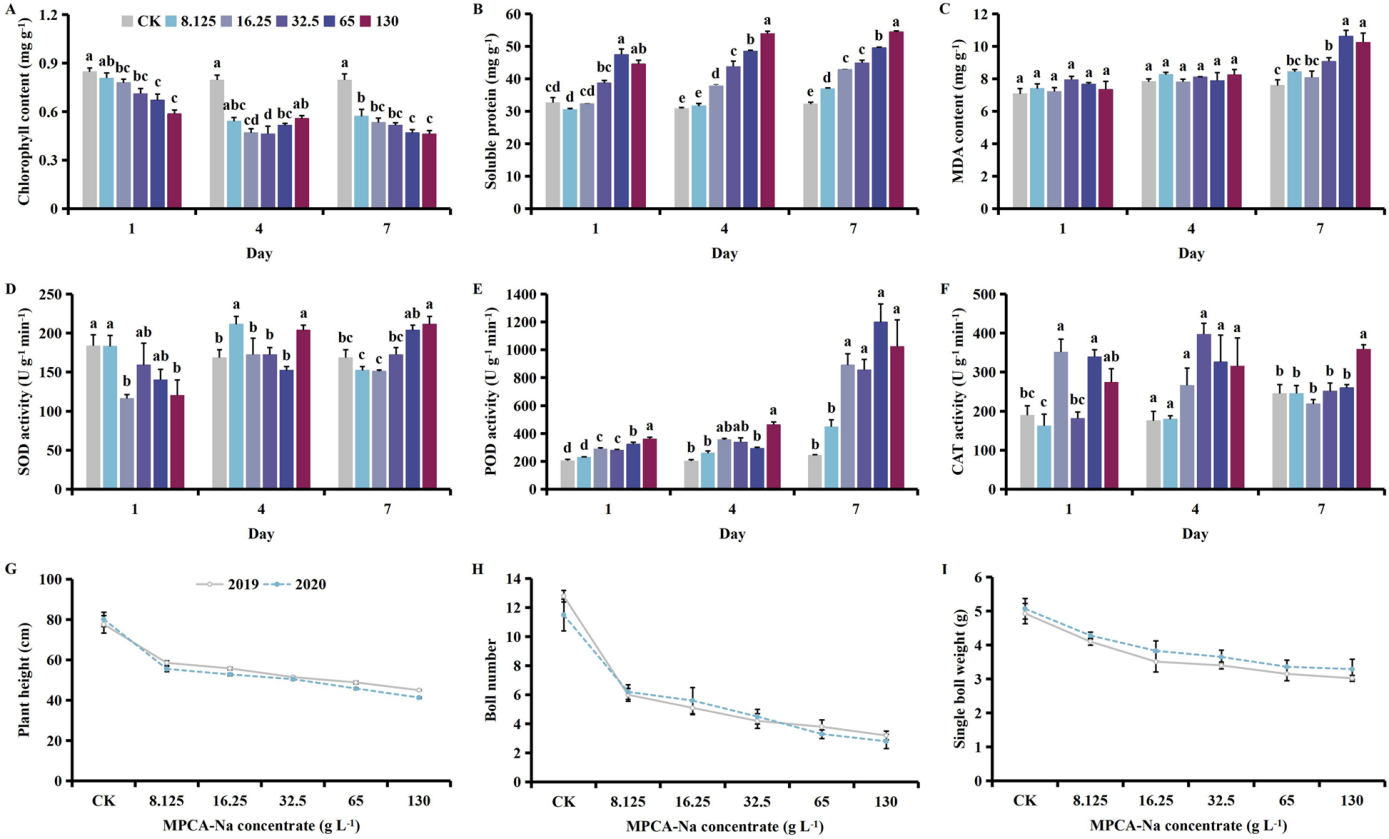
Effect of MCPA-Na exposure on the (**A**) chlorophyll, (**B**) soluble protein, and (**C**) MDA (malondialdehyde) content, (**D**) SOD (superoxide dismutase), (**E**) POD (peroxidase), (**F**) CAT (catalase) activities, (**G**) plant height, (**H**) boll number, and (**I**) the single boll weight at the seedling stage. Mean values ± standard error are shown (*p* < 0.05). Different lowercase letters indicate significant differences between treatments

MPCA-Na exposure at the seedling stage caused a decrease in chlorophyll, and increases in contents of soluble protein and MDA, and activity of all protective enzymes. Plant height, boll number and the single boll weight all decreased with increasing MPCA-Na (Fig. 1). Meanwhile, with time, the contents of chlorophyll and soluble protein decreased, contents of MDA and activities of SOD and POD increased, and activity of CAT decreased (Fig. S1).

Compared with the control, treatment with a high concentration of MPCA-Na (130 g L^-1^) caused decreases in the chlorophyll content of 30.72, 29.93, and 41.86% (*p* < 0.05) on days 1, 4 and 7 after treatment, respectively (Fig. 1A). Meanwhile, the soluble protein content increased by 34.62, 74.99 and 68.97%, while MDA level increased by 3.90, 5.43, and 34.95%, respectively (*p* < 0.05, Fig. 1B & C). The SOD activity decreased by 34.30%on day 1 after treatment then increased by 20.90 and 25.42% on days 4 and 7, respectively (*p* < 0.05, Fig. 1D). Meanwhile, POD activity increased by 76.28, 128.85, and 319.01%, while CAT activity increased by 44.68, 79.31, and 45.78% (*p* > 0.05), respectively (*p* > 0.05, Fig. 1E & F). Compared with the control, plant height decreased by 42.08%, the number of bolls decreased by 75.33%, and the single boll weight decreased by 46.42% following treatment with 130 g L^-1^ MPCA-Na (*p* < 0.05, Fig. 1G, H & I).

**Fig. 2:**
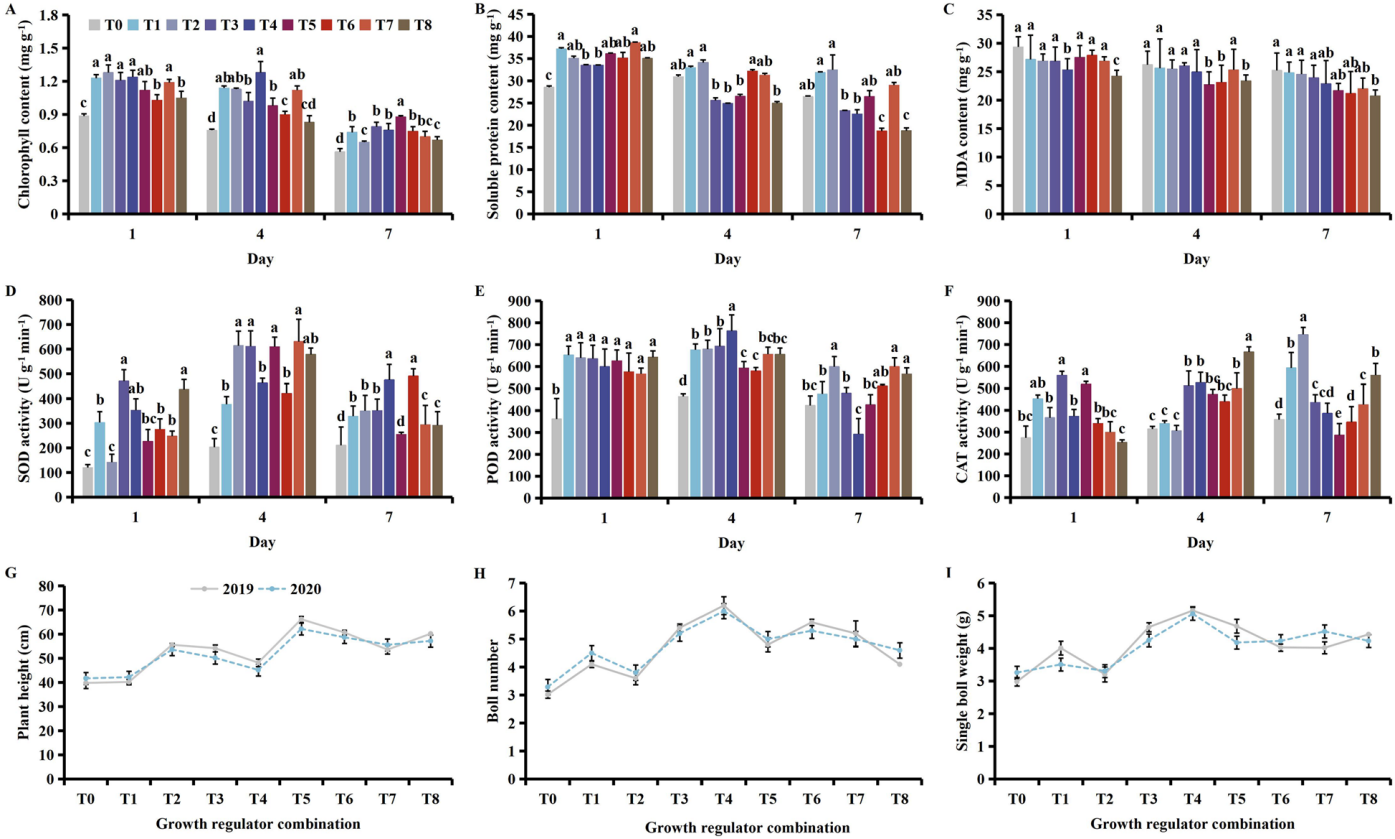
Effect of different combinations of plant growth regulators on the (**A**) chlorophyll, (**B**) soluble protein, (**C**) and MDA (malondialdehyde) content, (**D**) SOD (superoxide dismutase), (**E**) POD (peroxidase), (**F**) CAT (catalase) activities, (**G**) plant height, (**H**) boll number, and (**I**) the single boll weight following MCPA-Na exposure at the seedling stage. Mean values ± standard error are shown (*p* < 0.05) . Different lowercase letters indicate significant differences between treatments. T0 (control), T1 (brassinosteroids), T2 (gibberellin + seaweed fertilizer), T3 (brassinosteroids + seaweed fertilizer) and T4 (phthalanilic acid + seaweed fertilizer), T5 (gibberellin), T6 (brassinosteroids + gibberellin + phthalanilic acid + seaweed fertilizer), T7 (phthalanilic acid), T8 (brassinosteroids + gibberellin + phthalanilic acid)

Application of the plant growth regulators caused a decrease in the contents of chlorophyll, soluble protein and MDA, and had a positive effect on protective enzyme activity, and reducing cotton yield losses (Fig. 2). With time, the chlorophyll and soluble protein contents increased then decreased, while the MDA content showed a gradual increase, SOD and POD activity decreased, and CAT activity increased then decreased (Fig. S2).

On days 1 and 4, the chlorophyll content was significantly lower under T6 than T1, T2, T3 and T4 (Fig. 2A). Meanwhile, on days 1, 4 and 7, the soluble protein content was significantly lower under T3 and T4 than T1 (Fig. 2B). The MDA content was significantly lower under T8 than T1 (Fig. 2C), while on day 7, SOD activity was significantly higher under T4 and T6 than all other treatment groups (Fig. 2D). On day 4, highest POD activity was observed under T4 at 763.33 U g^− 1^  min^-1^ (Fig. 2E), while highest CAT activity was observed under T8 at 666.67 U g^− 1^  min^-1^ (Fig. 2F). Plant height was highest under T5 (Fig. 2G), while boll number and single boll weight were highest under T4 (Fig. 2H & I). It should be treated with phthalanilic acid + seaweed fertilizer at seedling stage to mitigate stress of MCPA-Na damage.

### Effects of MCPA-Na exposure and application of plant growth regulators at the budding stage

**Fig. 3:**
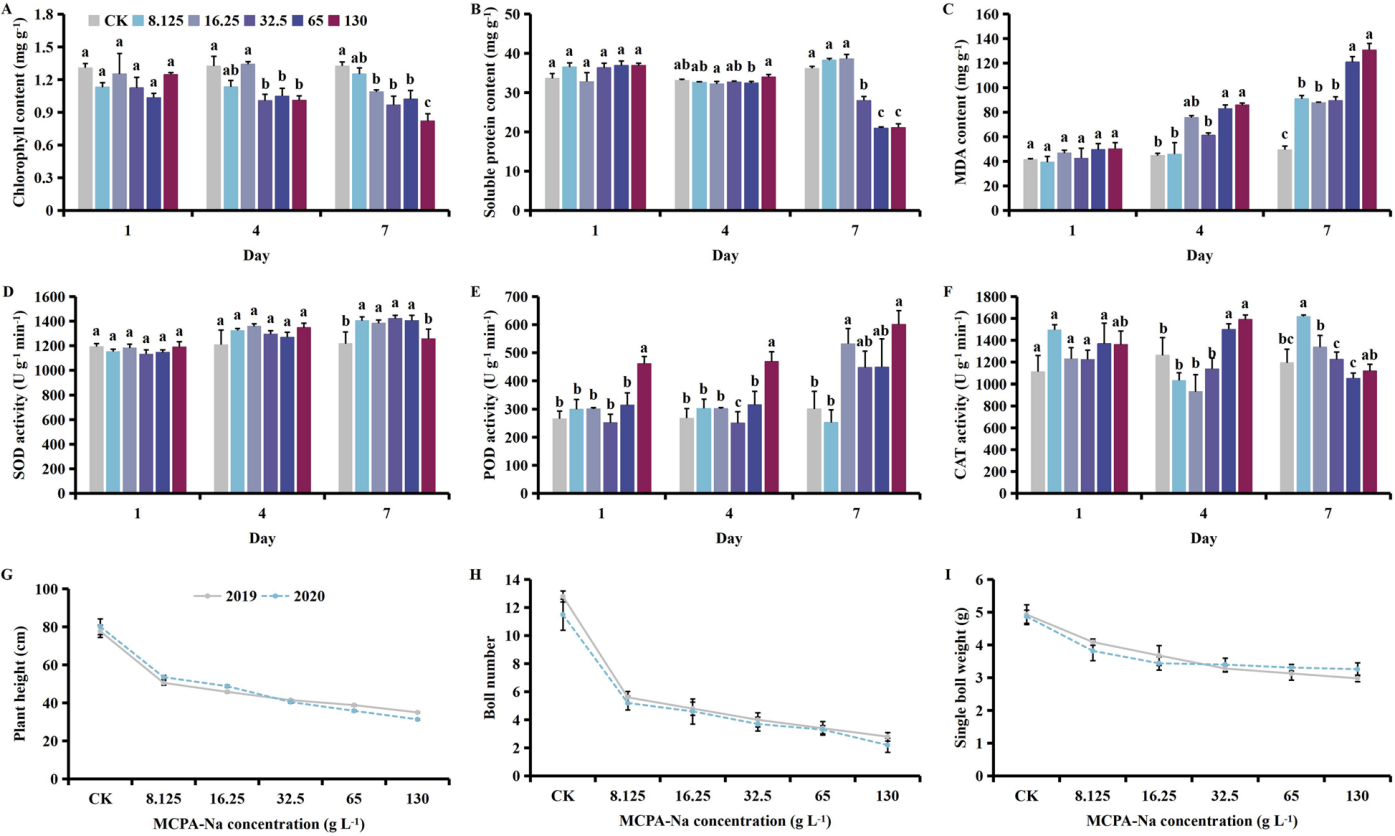
Effect of MCPA-Na exposure on the (**A**) chlorophyll, (**B**) soluble protein, and (**C**) MDA (malondialdehyde) content, (**D**) SOD (superoxide dismutase), (**E**) POD (peroxidase), (**F**) CAT (catalase) activities, (**G**) plant height, (**H**) boll number, and (**I**) the single boll weight at the budding stage. Mean values ± standard error are shown (*p* < 0.05). Different lowercase letters indicate significant differences between treatments

After MPCA-Na exposure at the budding stage, the content of chlorophyll decreased, while contents of soluble protein and MDA, and activity of the protective enzymes increased. Plant height, boll number and the single boll weight decreased with increasing MPCA-Na concentration (Fig. 3). With time, contents of chlorophyll and soluble protein gradually decreased, while the MDA content, and SOD and POD activities increased following treatment with 130 g L^-1^ MPCA-Na. CAT activity first increased then decreased (Fig. S3).

Compared with the control, the chlorophyll content decreased by 5.42, 23.68, and 38.07% on days 1, 4 and 7 after treatment with 130 g L^-1^ MPCA-Na, respectively (*p* < 0.05, Fig. 3A), while the soluble protein content increased by 9.75 and 2.53% on days 1 and 4 then decreased by 41.52% on day 7 (*p* < 0.05, Fig. 3B). The MDA content increased by 21.49, 92.10, and 164.19%, respectively (*p* > 0.05, Fig. 3C), while SOD activity decreased by 0.21% on day 1 then increased by 11.50 and 3.30% on days 4 and 7 (*p* < 0.05, Fig. 3D). POD activity increased by 73.32, 75.01, and 99.56%,while CAT activity increased by 22.32 and 25.95% on days 1 and 4 then decreased by 6.25% on day 7 (*p* < 0.05, Fig. 3E & F). Compared with the control, plant height decreased by 54.95%, the number of bolls decreased by 79.50%, and the single boll weight decreased by 36.31% following treatment with 130 g L^-1^ MPCA-Na (*p* < 0.05, Fig. 3G, H & I).

**Fig. 4:**
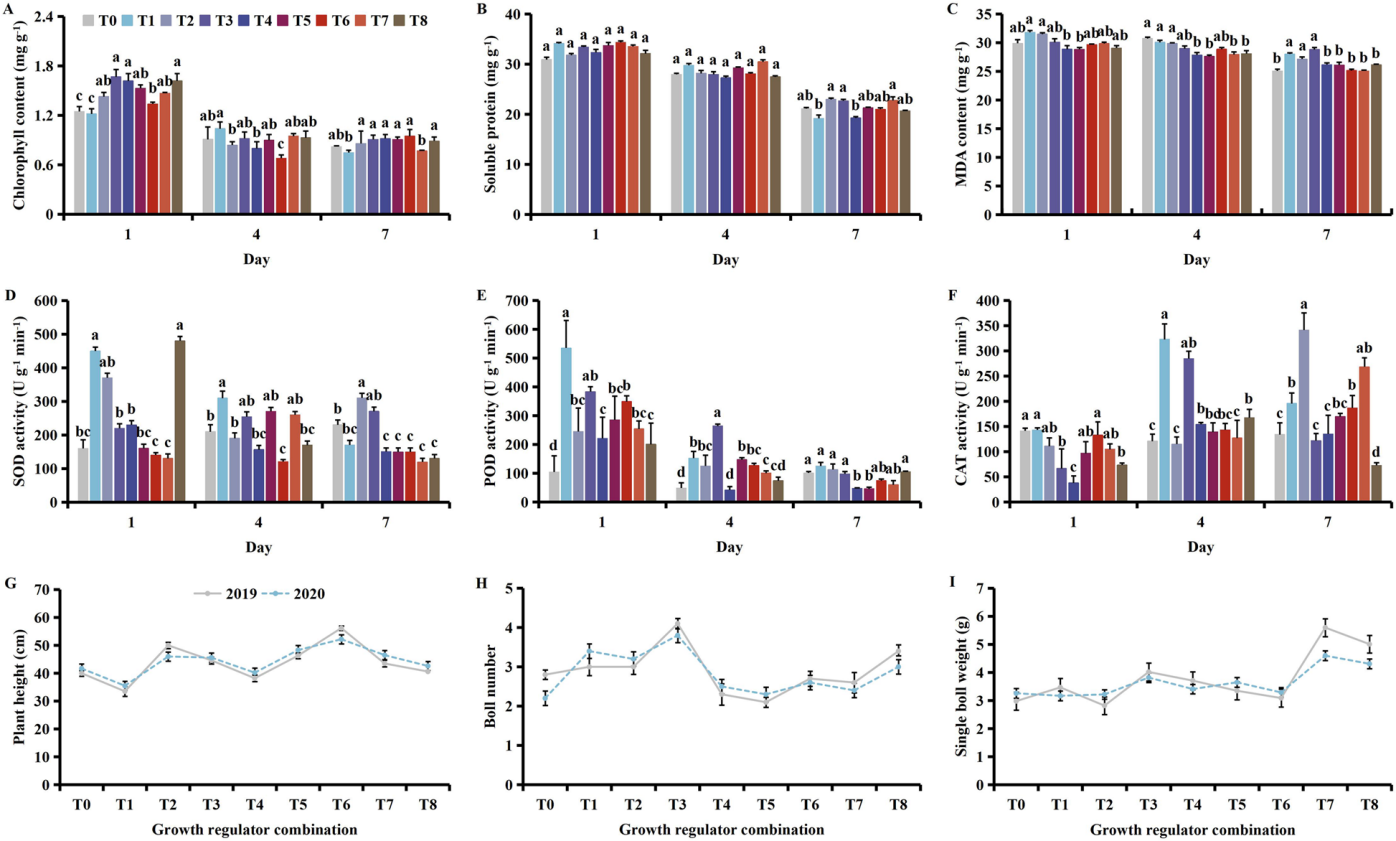
Effect of different combinations of plant growth regulators on the (**A**) chlorophyll, (**B**) soluble protein, and (**C**) MDA (malondialdehyde) content, (**D**) SOD (superoxide dismutase), (**E**) POD (peroxidase), (**F**) CAT (catalase) activities, (**G**) plant height, (**H**) boll number, and (**I**) the single boll weight following MCPA-Na exposure at the budding stage. Mean values ± standard error are shown (*p* < 0.05). Different lowercase letters the significant differences between treatments. T0 (control), T1 (brassinosteroids), T2 (gibberellin + seaweed fertilizer), T3 (brassinosteroids + seaweed fertilizer) and T4 (phthalanilic acid + seaweed fertilizer), T5 (gibberellin), T6 (brassinosteroids + gibberellin + phthalanilic acid + seaweed fertilizer), T7 (phthalanilic acid), T8 (brassinosteroids + gibberellin + phthalanilic acid)

Application of plant growth regulators increased the chlorophyll and soluble protein content, decreased the MDA content, and improved the protective enzyme activity, and reducing cotton yield losses (Fig. 4). With time, the chlorophyll, soluble protein, and MDA contents decreased along with POD activity, and improved SOD and CAT activity (Fig. S4).

On days 1 and 4, the chlorophyll content was significantly higher under T2, T3, T4 and T8 than T6 (Fig. 4A). On day 7, the soluble protein content was highest under T2 at 23.0 mg g^-1^ (Fig. 4B), while on days 1, 4 and 7, the MDA content was significantly lower under T4 and T5 than under T1 and T2 (Fig. 4C). On day 1, SOD activity was highest under T8 at 48.75 U g^− 1^  min^-1^ (Fig. 4D), while POD activity was highest under T1 at 535.35 U g^− 1^  min^-1^ (Fig. 4E). On day 7, CAT activity was highest under T2 at 341.41 U g^− 1^  min^-1^ (Fig. 4F). Plant height was highest under T6 (Fig. 4G), while the number of cotton bolls was highest under T3 (Fig. 4H), and the single boll weight was highest under T7 (Fig. 4I). It should be treated with 24-epibrassinolide + seaweed fertilizer at buddling stage to mitigate stress of MCPA-Na damage.

### Effects of MCPA-Na exposure and application of plant growth regulators at the flowering and boll stages

**Fig. 5:**
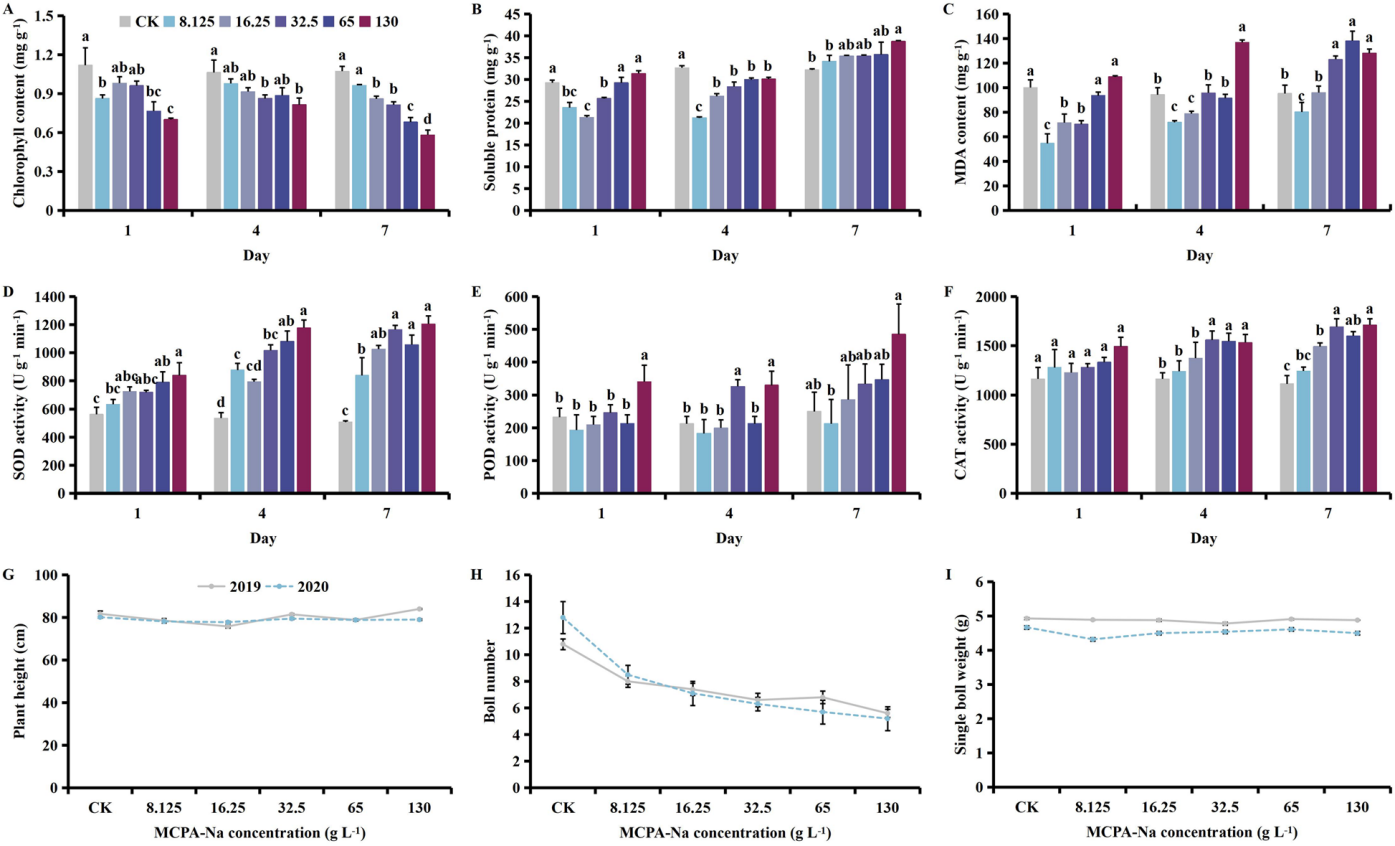
Effect of MCPA-Na exposure on the (**A**) chlorophyll, (**B**) soluble protein, and (**C**) MDA (malondialdehyde) content, (**D**) SOD (superoxide dismutase), (**E**)POD (peroxidase), (**F**) CAT (catalase) activities, (**G**) plant height, (**H**) boll number, and (**I**) the single boll weight at the flowering and boll stages. Mean values ± standard error are shown (*p* < 0.05). Different lowercase letters indicate significant differences between treatments

MPCA-Na exposure decreased the chlorophyll content, increased the soluble protein content, MDA content and protective enzyme activity, had little effect on plant height and boll weight, and reduced the boll number at both the flowering and boll stages (Fig. 5). Meanwhile, with time, the chlorophyll content decreased, while the soluble protein content, MDA content and protective enzyme activity increased (Fig. S5).

Compared with the control, the chlorophyll content under 130 g L^-1^ MPCA-Na decreased by 37.37, 23.39, and 45.78% on days 1, 4 and 7, respectively (*p* < 0.05, Fig. 5A), while the soluble protein content decreased by 7.87% on day 4 then increased by 7.25 and 20.31% on days 1 and 7, respectively (*p* < 0.05, Fig. 5B). The MDA content through different treatments increased by 8.74, 45.15 and 34.34%, respectively at *p* < 0.05 (Fig. 5C). Meanwhile, SOD activity increased by 48.91, 119.85, and 137.03%, POD activity increased by 45.71, 54.69, and 93.62%, and CAT activity increased by 28.44, 31.81, and 53.89%, respectively (*p* < 0.05, Fig. 5D, E & F). Plant height increased by 2.82%, boll number decreased by 48.15%, and the single boll weight decreased by 5.38% (*p* > 0.05, Fig. 5G, H & I).

**Fig. 6:**
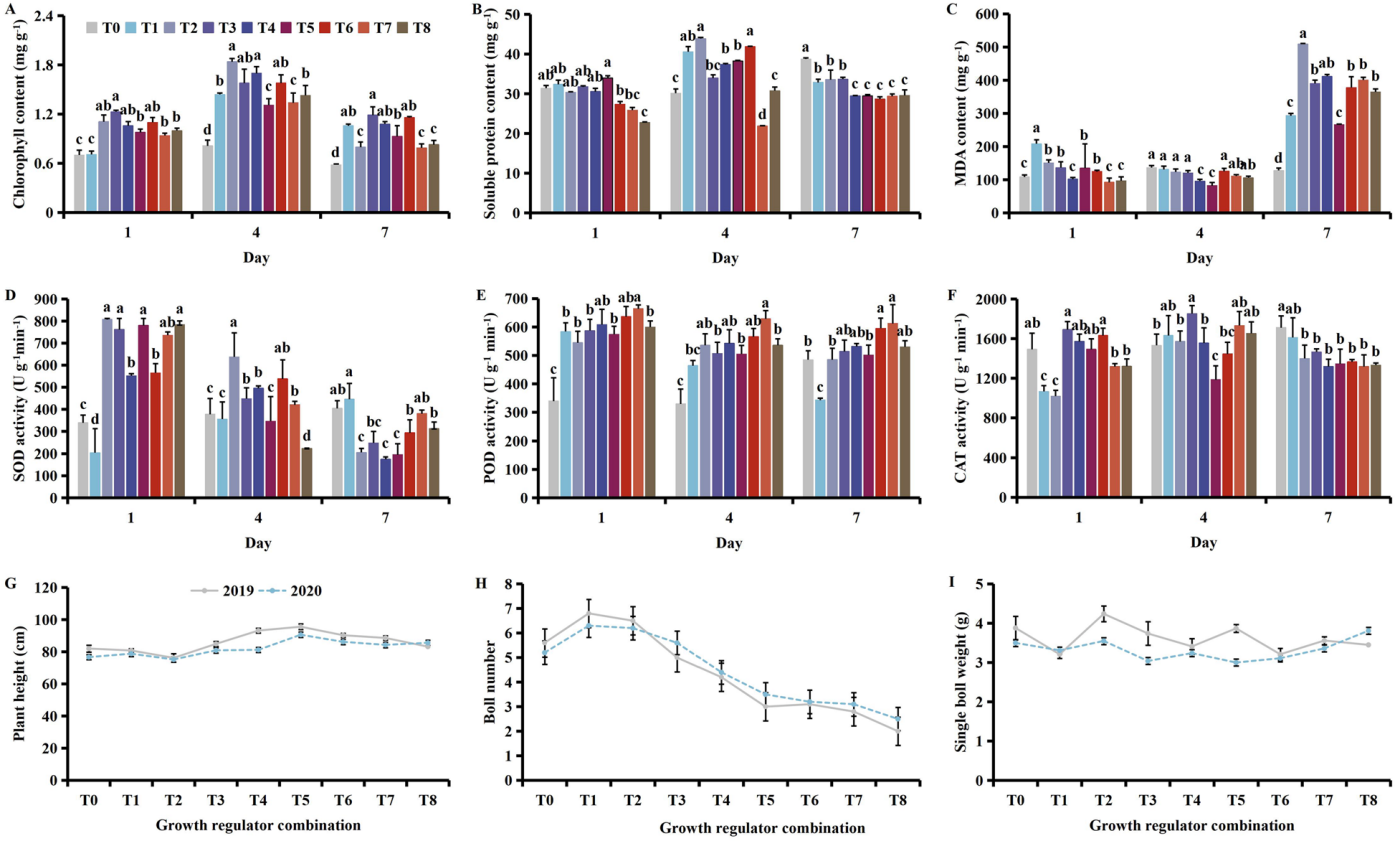
Effect of different combinations of plant growth regulators on the (**A**) chlorophyll, (**B**) soluble protein, and (**C**) MDA  (malondialdehyde) content, (**D**) SOD (superoxide dismutase), (**E**) POD (peroxidase), (**F**) CAT (catalase) activities, (**G**) plant height, (**H**) boll number, and (**I**) the single boll weight following MCPA-Na exposure at the flowering and boll stages. Mean values ± standard error are shown (*p* < 0.05). Different lowercase letters indicate significant differences between treatments. T0 (control), T1 (brassinosteroids), T2 (gibberellin + seaweed fertilizer), T3 (brassinosteroids + seaweed fertilizer) and T4 (phthalanilic acid + seaweed fertilizer), T5 (gibberellin), T6 (brassinosteroids + gibberellin + phthalanilic acid + seaweed fertilizer), T7 (phthalanilic acid), T8 (brassinosteroids + gibberellin + phthalanilic acid)

Application of the plant growth regulators increased the protective enzyme activity, reduced the MDA content, and stabilized plant height and the single boll weight (Fig. 6). With time, the chlorophyll and soluble protein content increased then decreased (except under T7), while the MDA content gradually increased, SOD (except under T1) and POD activity decreased, and CAT activity gradually increased under T1 only (Fig. S6).

On day 4, the chlorophyll and soluble protein contents were highest under T2 at 1.84 and 4.39 mg g^-1^, respectively (Fig. 6A & B), while the MDA content was significantly lower under T5 than T1 (Fig. 6C). CAT activity was highest under T3 at 1853.33 U g^− 1^  min^-1^ (Fig. 6F), while on day 1, SOD activity was highest under T2 at 808.00 U g^− 1^  min^-1^ (Fig. 6D) and POD activity was highest under T7 at 664.83 U g^− 1^  min^-1^ (Fig. 6E). Plant height was highest under T5 (Fig. 6G), the number of cotton bolls was highest under T1 (Fig. 6H), and the single boll weight was highest under T2 (Fig. 6I). It should be treated with GA_3_ + seaweed fertilizer at flowering and boll stage to mitigate stress of MCPA-Na damage.

## Discussion

Most herbicides have injurious effects on crop growth, causing leaves to shrink, inhibiting plant height, and causing flowers and fruit to drop, all of which cause serious reductions in crop yield [31, 32]. After exposure, crops tend to regulate their physiological and metabolic functions in order to convert herbicides into harmless compounds [33]. However, not all herbicides can be metabolized. For example, MCPA-Na is not metabolized by cotton [6], therefore such plants need to actively regulate their physiological metabolism in order to reduce subsequent damage. The regulatory mechanisms underlying protective enzyme responses to damage have been confirmed in a number of plants [34]. For example, 2, 4-D was found to cause an increase in protective enzyme activity in wheat and rice [35, 36], while MCPA causes an increase in protective enzyme activity in millet [37], and MCPA-Na induces an increase in bloom-forming cyanobacteria protective enzyme activity [38]. In this study, MCPA-Na exposure at different growth stages caused increases in the soluble protein content and protective enzyme activity, and decreases in the chlorophyll content. These findings suggest that cotton does not minimize MCPA-Na damage by enhancing photosynthesis, but rather by increasing basal metabolism and protective enzyme activity.

The growth period of plants determines their sensitivity to herbicides, as shown by the sensitivity of cotton, cucumber and watermelon to 2, 4-D exposure [39–41]. In this study, resistance of cotton to MCPA-Na at different growth stages was also found to differ. For example, at the seedling stage, MDA began to accumulate on day 7 after exposure, while at the budding stage, accumulation began on day 4. Moreover, at the flowering and boll stages, the MDA content was lower than the control on days 1, 4 and 7 at MCPA-Na concentrations of 8.125 and 16.25 g L^-1^ MCPA-Na. Meanwhile, plant height and the single boll weight were less affected by MCPA-Na damage at the flowering and boll stages, but together with boll number were significantly affected at the seedling and budding stages. These findings suggest that resistance of cotton to MCPA-Na is stronger at the flowering and boll stages than at the seedling and budding stages. In other words, the vegetative growth phase is more sensitive to MCPA-Na toxicity.

The limited defense capacity of plants is unable to provide sustainable or high-level metabolic responses during exposure to herbicides that are slowly metabolized or even unable to be metabolized in plants [42, 43]. Moreover, with increased exposure, plants are more prone to serious damage or even death [44, 45]. When multiple reactive oxygen species cannot be eliminated by the antioxidant enzyme system, oxidative stress and lipidization of the cell membrane are induced, resulting in an accumulation of MDA in vivo [46, 47]. In this study, the increased exposure to MCPA-Na, notably on day 7, caused an increase in MDA accumulation, which was not eliminated by normal metabolism. This finding suggests that the defense responses of cotton are unable to cope with prolonged MCPA-Na damage, highlighting the need for additional measures to enhance the defense ability and reduce damage.

Plant growth regulators are often used to alleviate herbicide damage [48]. For example, brassinosteroids were found to protect maize from amphetamine damage [21], while gibberellin can alleviate S-metolachlor damage in rice seedlings [23]. In general, plant growth regulators help plants increase their metabolic levels, including antioxidant capacity and related gene expression [49–53], and reducing herbicide damage [54, 55]. In this study, combined application of plant growth regulators improved physiological metabolism in cotton exposed to MCPA-Na, increasing the chlorophyll content and reducing the MDA content. It is worth noting that specific plant growth regulators should be used in different growth stages of cotton, because the demand for plant growth regulators is different in different growth stages of cotton [52, 53], and the resistance of cotton to herbicide damage is also different. From a physiological point of view, the application of phthalanilic acid + seaweed fertilizer (T4) alleviated MCPA-Na damage at the seedling stage, the application of 24-epibrassinolide + seaweed fertilizer (T3) alleviated MCPA-Na damage at the budding stage, while at the flowering and boll stage, the application of GA_3_ + seaweed fertilizer (T2) is recommended.

There are antagonistic or synergistic effects between different kinds of plant growth regulators. It is necessary to use them reasonably according to the characteristics of their effects to reduce herbicide damage. 24-epibrassinolide can effectively improve the chlorophyll content and photosynthetic efficiency of plants [56, 57], protect flowers and fruits [58], and reduce the occurrence of drug damage [59]. GA_3_ can promote plant flowering [60], increase fruit setting rate and yield [61]. Phthalanilic acid can promote the transport of nutrients to growth points such as buds, enhance plant cell viability, promote chlorophyll synthesis, and have a synergistic effect with auxin [62, 63]. As a natural seaweed extract, seaweed fertilizer has the dual functions of plant regulation and nutrition supplementation [64], promoting root development and improving absorption and utilization, and can also protect flowers and fruits and improve fruit yield and quality [65, 66]. Therefore, in this study, we used the characteristics of different kinds of plant growth regulators, and combined these plant growth regulators in different damage stages of cotton. In the seedling stage, after being damaged by MCPA-Na, the combination of phthalanilic acid and seaweed fertilizer can synergistically promote the vegetative growth of cotton, promote the synthesis of chlorophyll and root development, improve the absorption and utilization of nutrients and antioxidant capacity, and can better resist MCPA-Na. In the budding stage, the synergistic effect of 24-epibrassinolide and seaweed fertilizer can protect cotton buds, improve photosynthesis and antioxidant capacity, and promote reproductive growth. In the flowering and boll stage, the synergistic effect of GA_3_ and seaweed fertilizer can promote the boll setting of cotton and increase the yield. We suggest that when using plant growth regulators to alleviate the phytotoxicity of cotton, it should be used in a scientific combination according to the characteristics of cotton growth stage and plant growth regulators, so as to avoid antagonism and secondary phytotoxicity when using growth regulators.

Under a fixed planting density, cotton yield is determined by the number of bolls and the single boll weight [67]. Herbicide damage tends to reduce both traits, thereby reducing yield [68–70]. A recent study also found that cotton yield is differentially affected by different herbicides at different growth stages [39]. For example, 2, 4-D was found to have differential effects on the single boll weight and boll number at different growth stages [39]. In this study, MCPA-Na affected cotton yield by reducing the boll number and single boll weight at the seedling and budding stages, and by reducing the boll number at the flowering and boll stage. Compared with the seedling and budding stage, the effect on yield is relatively small at the flowering and boll stage that exposure by MCPA-Na. Meanwhile, the application of plant growth regulators caused an increase in the single boll weight and boll number following MCPA-Na exposure.

In terms of yield, the findings of this study suggest that cotton exposed to MCPA-Na at the seedling stage should be treated with phthalanilic acid + seaweed fertilizer, while plants exposed at the budding stage should be treated with 24-epibrassinolide + seaweed fertilizer, and those exposed at the flowering and boll stage should be treated with GA_3_ + seaweed. However, despite these findings, the effect of the plant growth regulators on cotton damaged at the flowering and boll stage was less than satisfactory, causing branches and leaves lush and boll opening difficulties at later stages. Therefore, for cotton exposed at these later stages, we suggest a low dosage of plant growth regulators to avoid shoot elongation and reduce the impact on yield.

## Conclusions

Overall, this study clarified the physiological and metabolic response of cotton plants to MCPA-Na exposure at different growth stages, as well as the differing effects on plant height and subsequent yield. Moreover, the mitigating effects of different combinations of plant growth regulators were also confirmed at different growth stages. Following MCPA-Na exposure, the soluble protein content and protective enzymes activity played an important role in the defense response of cotton. The single boll weight and boll number decreased significantly at the seedling and budding stages, while only the boll number decreased significantly at the flowering and boll stages, suggesting that the vegetative growth stage is more sensitive to MCPA-Na damage. Meanwhile, the application of plant growth regulators reduced the content of MDA, helping reduce MCPA-Na damage, and they also had a positive effect on the cotton yield traits. These findings suggest that in production practice, plant growth regulators could be reasonably applied to alleviate MCPA-Na damage in the field; however, more importantly, the drift of herbicides should be avoided, and the occurrence of pesticide accidents should be fundamentally reduced.

## Supplementary Information

**Additional file 1.**

## Data Availability

The raw data of the presented results of this study are available on request to the corresponding author.

## Abbreviations

2-methyl-4-chlorophenoxy acetic acid-Na: MPCA-Na
POD: Peroxidase
CAT: Catalase
SOD: Superoxide dismutase
MDA: Malondialdehyde
