# Supplementary material for: Use of plant growth regulators to reduce 2-methyl-4-chlorophenoxy acetic acid-Na (MPCA-Na) damage in cotton (Gossypium hirsutum)
Source: BMC Plant Biol. 2022 Nov 16;22:533. doi: 10.1186/s12870-022-03917-x (PMC9667669; doi:10.1186/s12870-022-03917-x)
Supplement: Supplementary file 1 — Additional file 1. [file 12870_2022_3917_MOESM1_ESM.pdf]

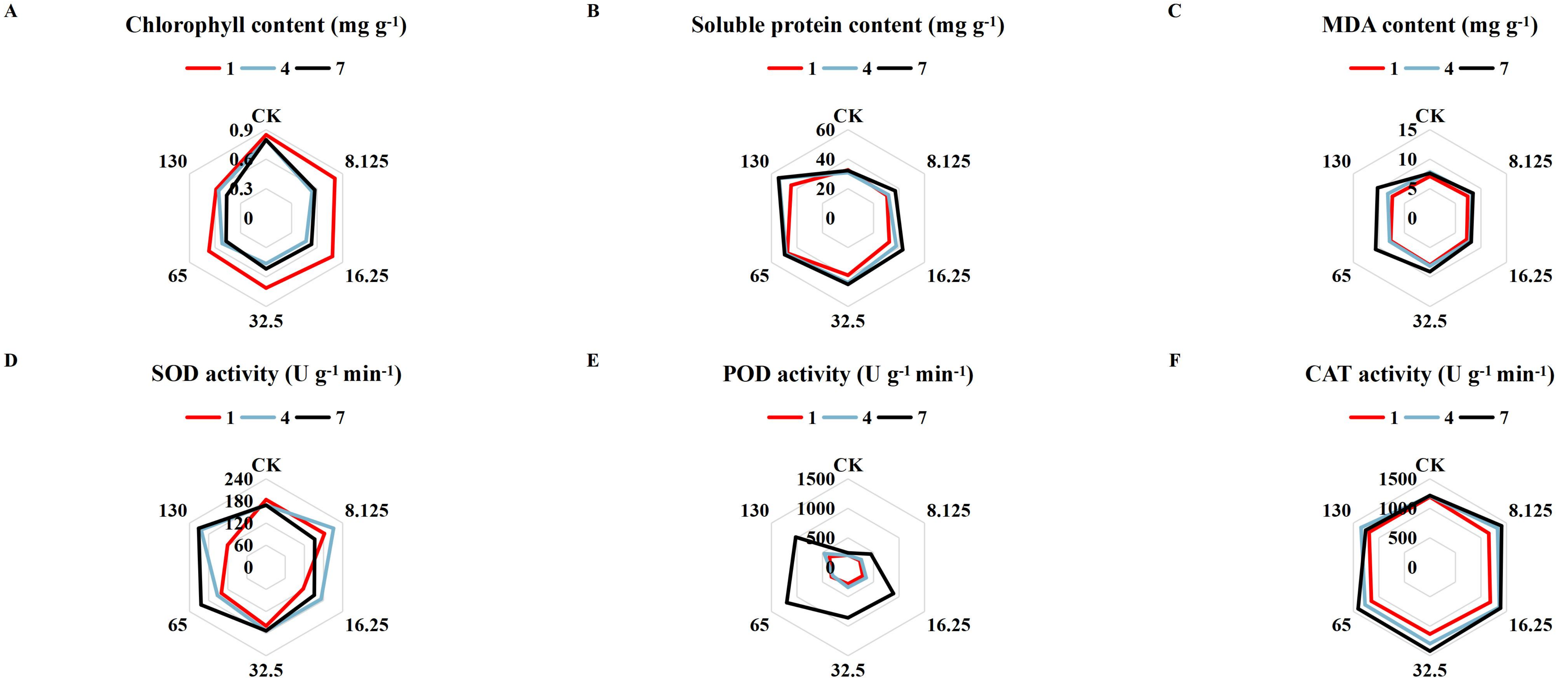

Fig. S1 Effect of 2-methyl-4-chlorophenoxy acetic acid-Na (MCPA-Na) exposure with time on the (**A**) chlorophyll, (**B**) soluble protein, and (**C**) MDA (malondialdehyde) content, and (**D**) SOD (superoxide dismutase), (**E**) POD (peroxidase), (**F**) CAT (catalase) activities at the seedling stage.

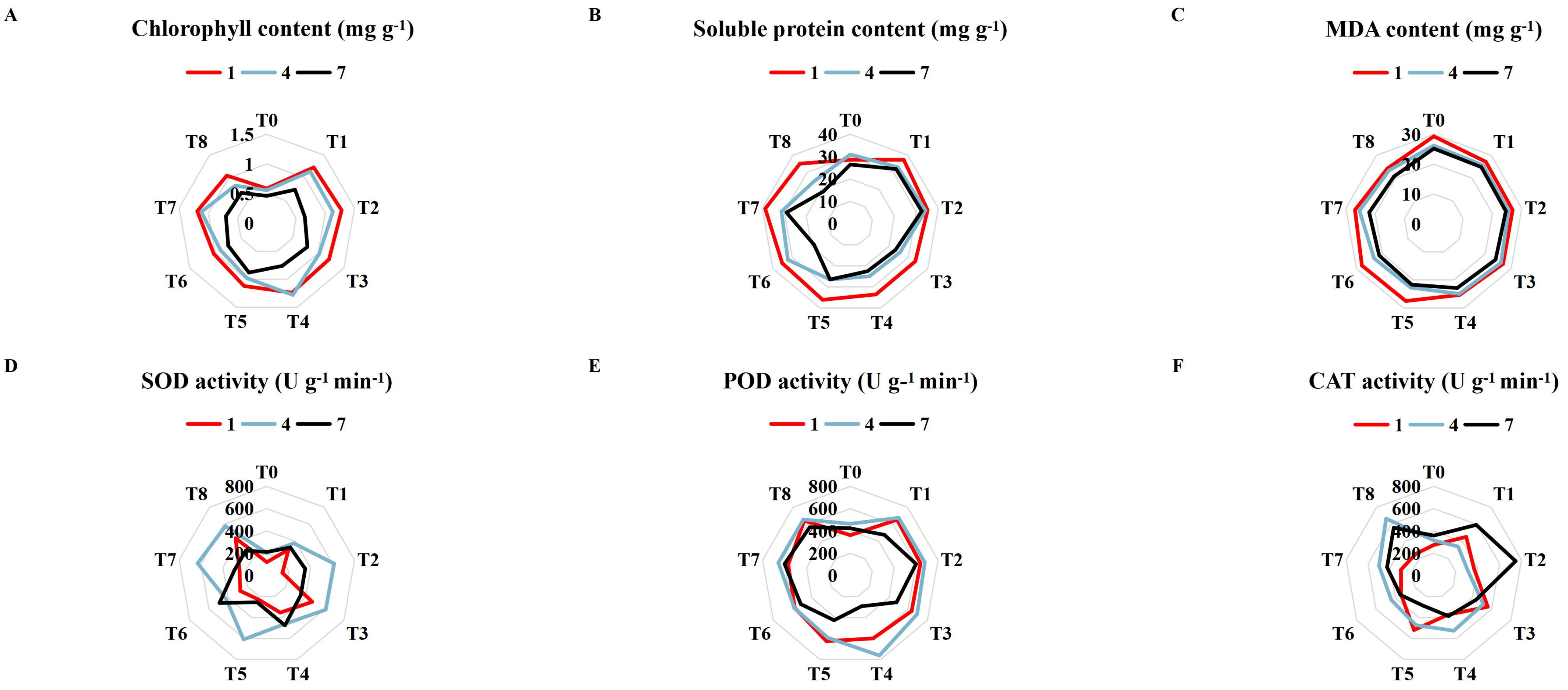

Fig. S2 Effect of different combinations of plant growth regulators with time on the (**A**) chlorophyll, (**B**) soluble protein, and (**C**) MDA (malondialdehyde) content, and (**D**) SOD (superoxide dismutase), (**E**) POD (peroxidase), (**F**) CAT (catalase) activities at the seedling stage. T0 (control), T1 (brassinosteroids), T2 (gibberellin + seaweed fertilizer), T3 (brassinosteroids + seaweed fertilizer) and T4 (phthalanilic acid + seaweed fertilizer), T5 (gibberellin), T6 (brassinosteroids + gibberellin + phthalanilic acid + seaweed fertilizer), T7 (phthalanilic acid), T8 (brassinosteroids + gibberellin + phthalanilic acid).

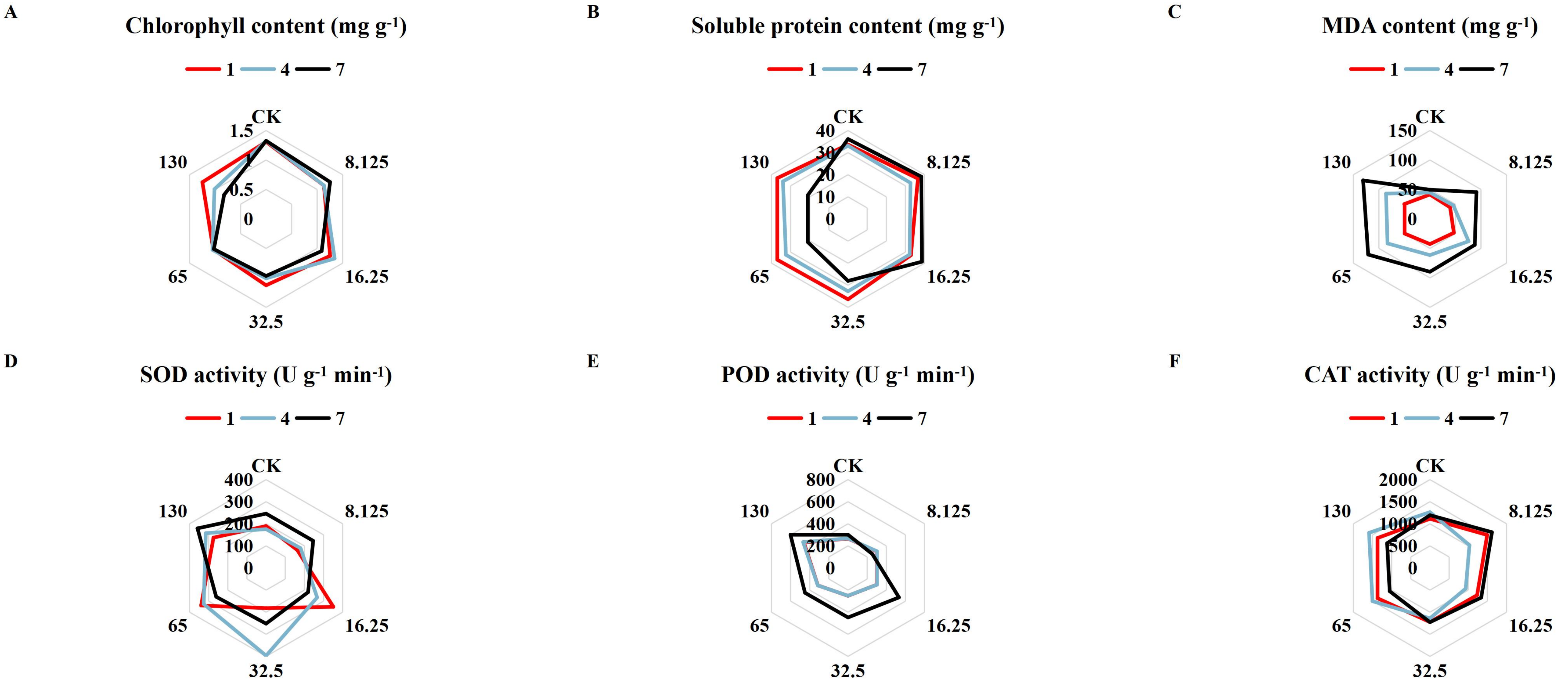

Fig. S3 Effect of 2-methyl-4-chlorophenoxy acetic acid-Na (MCPA-Na) exposure with time on the (**A**) chlorophyll, (**B**) soluble protein, and (**C**) MDA (malondialdehyde) content, and (**D**) SOD (superoxide dismutase), (**E**) POD (peroxidase), (**F**) CAT (catalase) activities at the budding stage.

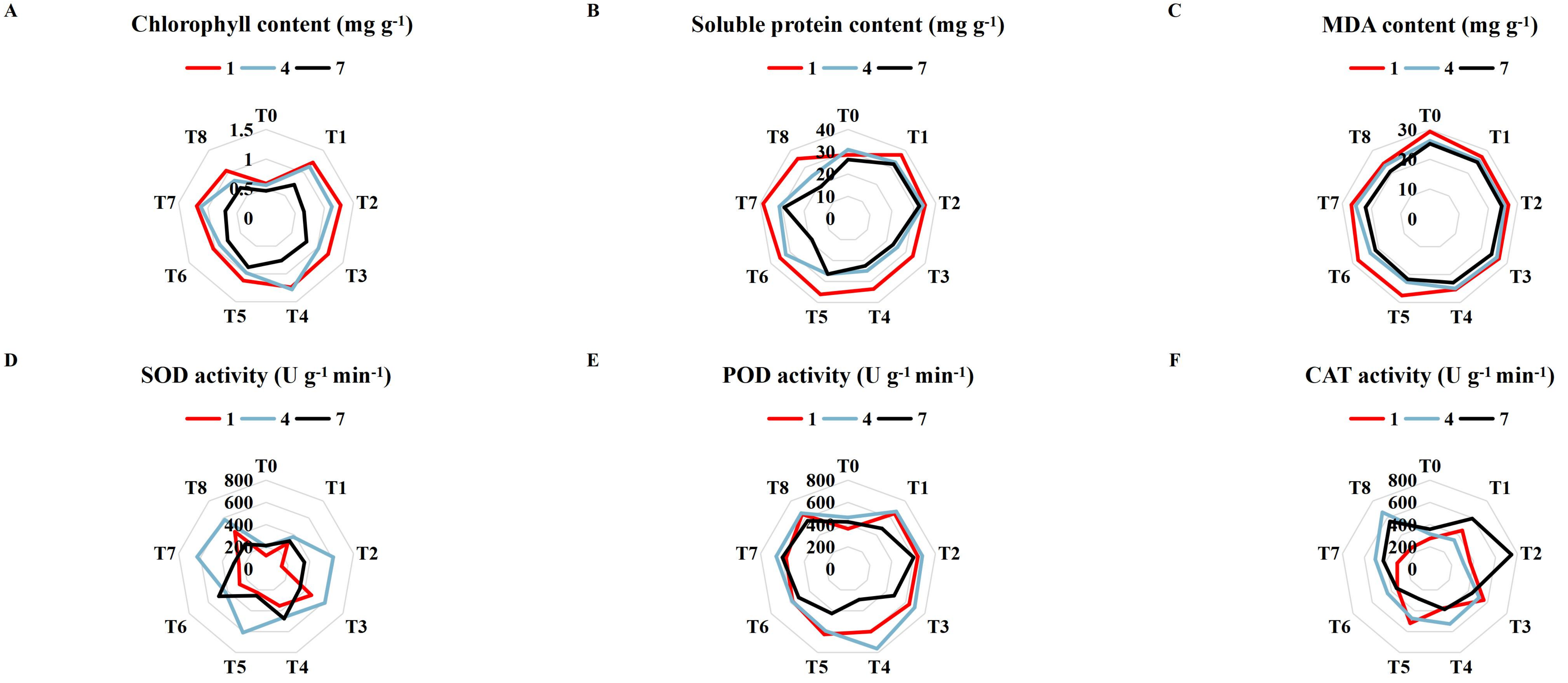

Fig. S4 Effect of different combinations of plant growth regulators with time on the (**A**) chlorophyll, (**B**) soluble protein, and (**C**) MDA (malondialdehyde) content, and (**D**) SOD (superoxide dismutase), (**E**) POD (peroxidase), (**F**) CAT (catalase) activities at the budding stage. T0 (control), T1 (brassinosteroids), T2 (gibberellin + seaweed fertilizer), T3 (brassinosteroids + seaweed fertilizer) and T4 (phthalanilic acid + seaweed fertilizer), T5 (gibberellin), T6 (brassinosteroids + gibberellin + phthalanilic acid + seaweed fertilizer), T7 (phthalanilic acid), T8 (brassinosteroids + gibberellin + phthalanilic acid).

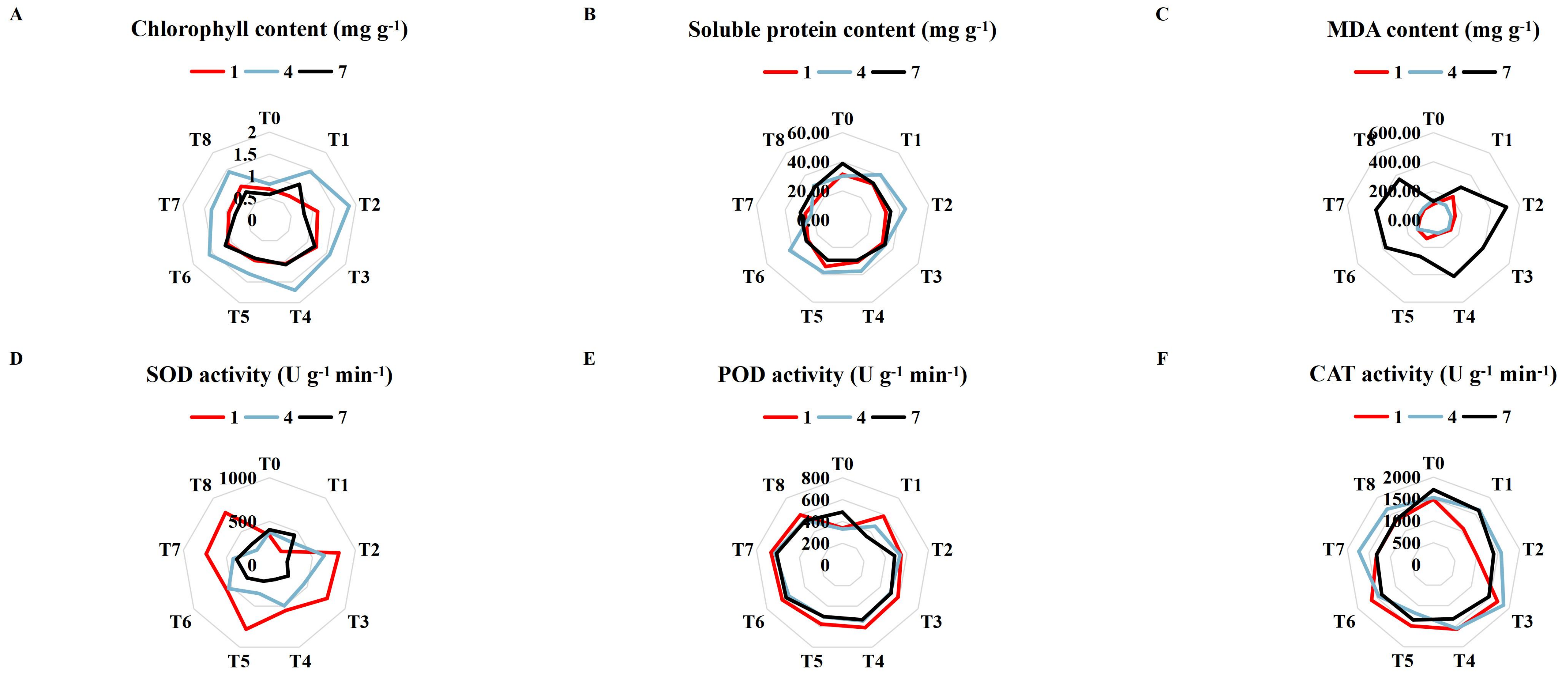

Fig. S5 Effect of 2-methyl-4-chlorophenoxy acetic acid-Na (MCPA-Na) exposure with time on the (**A**) chlorophyll, (**B**) soluble protein, and (**C**) MDA (malondialdehyde) content, and (**D**) SOD (superoxide dismutase), (**E**) POD (peroxidase), (**F**) CAT (catalase) activities at the flowering and boll stages.

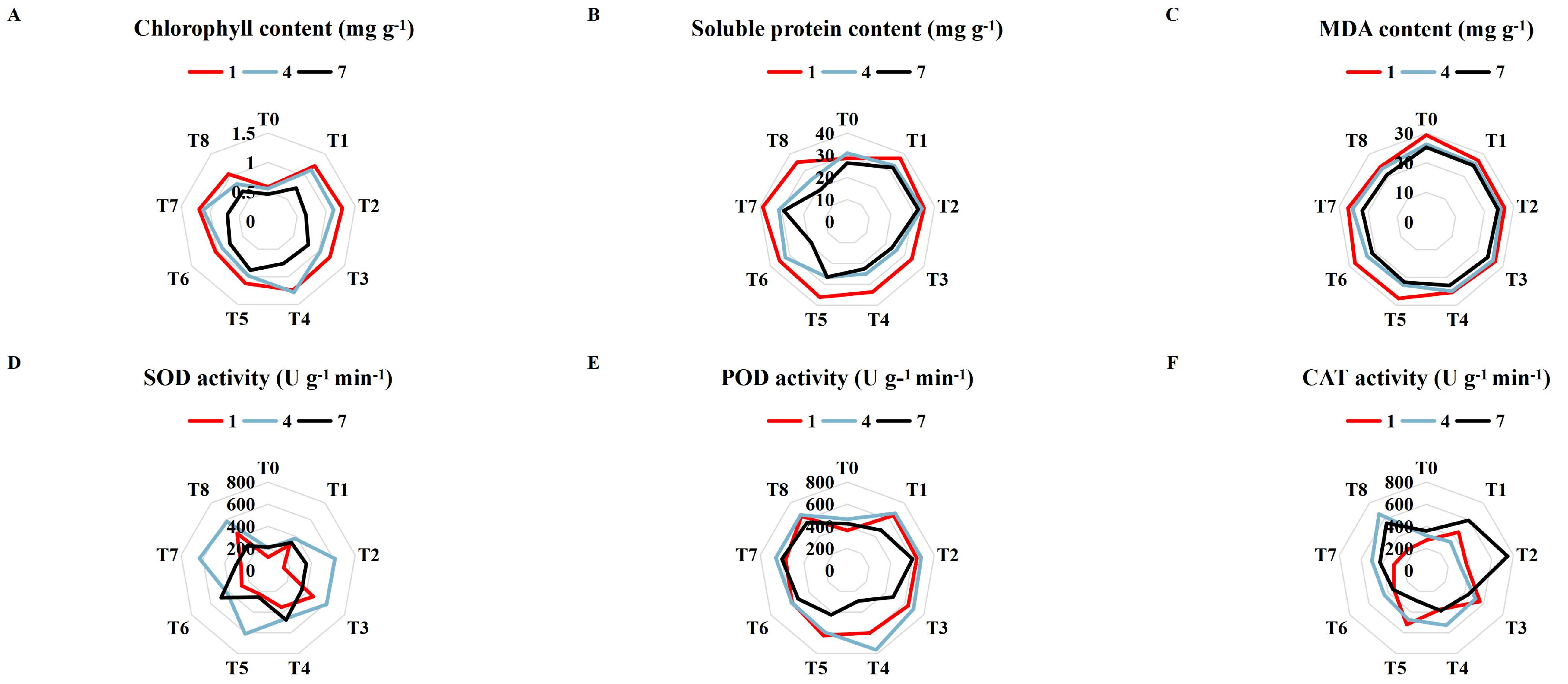

Fig. S6 Effect of different combinations of plant growth regulators with time on the (**A**) chlorophyll, (**B**) soluble protein, and (**C**) MDA (malondialdehyde) content, and (**D**) SOD (superoxide dismutase), (**E**) POD (peroxidase), (**F**) CAT (catalase) activities at the flowering and boll stages. T0 (control), T1 (brassinosteroids), T2 (gibberellin + seaweed fertilizer), T3 (brassinosteroids + seaweed fertilizer) and T4 (phthalanilic acid + seaweed fertilizer), T5 (gibberellin), T6 (brassinosteroids + gibberellin + phthalanilic acid + seaweed fertilizer), T7 (phthalanilic acid), T8 (brassinosteroids + gibberellin + phthalanilic acid).
